# A Systems Biology Starter Kit for Arenaviruses

**DOI:** 10.3390/v4123625

**Published:** 2012-12-11

**Authors:** Magali E. Droniou-Bonzom, Paula M. Cannon

**Affiliations:** Department of Molecular Microbiology and Immunology, Keck School of Medicine, University of Southern California, 2011 Zonal Avenue, Los Angeles, CA 90 033, USA; E-Mail: pcannon@usc.edu

**Keywords:** Arenavirus, interactome, infectome, virhostome, biological networks, systems biology, Lassa fever virus, Pichinde virus, Lymphocytic choriomeningitis virus, Junin virus

## Abstract

Systems biology approaches in virology aim to integrate viral and host biological networks, and thus model the infection process. The growing availability of high-throughput “-omics” techniques and datasets, as well as the ever-increasing sophistication of *in silico* modeling tools, has resulted in a corresponding rise in the complexity of the analyses that can be performed. The present study seeks to review and organize published evidence regarding virus-host interactions for the arenaviruses, from alterations in the host proteome during infection, to reported protein-protein interactions. In this way, we hope to provide an overview of the interplay between arenaviruses and the host cell, and lay the foundations for complementing current arenavirus research with a systems-level approach.

## 1. Introduction

### 1.1. Systems Biology as a Tool for Virus Research

Cells can be considered as complex circuits of interlinked molecular processes, whose activation or deactivation depends on stimulus-sensing, signaling, and pathway regulation through feedback loops. These processes and relationships can also be described mathematically in biological networks, using the concepts of graph theory, where cellular components such as proteins are represented as nodes or vertices, and biological relationships, for example protein-protein interactions, are indicated by connectors or edges. Recent technical advances that have allowed high-throughput and multivariate investigations of biological processes have generated a wealth of data about relationships in the cell that lends itself to such analyses. Systems biology refers to the study of how interactions between the components of a biological system give rise to the functions and behavior of that system, and can therefore be conceptually defined as a holistic approach to the investigation of biological processes.

Virus infection imposes new variables on the cell circuitry. On the one hand, viral components or ‘patterns’ are sensed by the infected cell, leading to the activation of distinct signaling cascades and alterations in cellular gene expression. In addition, the virus itself specifically targets cellular pathways as it attempts to deflect anti-viral attacks, and subvert the cellular machinery to complete its replication cycle. Such virus-induced modifications thus amount to a re-wiring of the biological circuitry of the host [1,2]. Of note, this re-wiring is often achieved by the virus targeting specific ‘hubs’ within the cell, defined as proteins with many interacting partners and/or that are central to several pathways, and which are identified in network analyses by parameters of connectivity and centrality [3,4]. In this way, even a small number of viral proteins can impact a large number of host processes and produce a complex phenotype that may be difficult to interpret without a systems-level approach. 

As a practical consequence of these types of analyses, the modeling of virus-induced perturbations of the host network can be used to identify potential viral targets or hubs, which can then be experimentally tested. For example, network analysis predicted that enoyl-coA isomerase activity was important for HCV infection [5], and this was subsequently confirmed experimentally [6]. Similarly, networks can be used to predict additional consequences of any experimentally observed virus-host interaction, which may in turn provide an explanation for other observed pathogenic effects. In Coxsackie virus B3 infection, the contribution of an autocrine feedback circuit involving the proinflammatory cytokines TNF and IL-1 to virus-induced myocardial damage was initially predicted through network analysis [7]. Ultimately, identifying the most basic or direct levels of the host-virus interface in this way can suggest important targets for the development of antiviral therapies.

### 1.2. The Arenaviruses and Studies of their Impact on the Host Cell

The arenaviruses express only 4 proteins: an RNA-dependent RNA polymerase (L) that carries out viral RNA synthesis, a matrix protein (Z) that drives viral budding, a nucleoprotein (NP) that encapsidates viral RNA, and a glycoprotein (GP) that mediates entry. Similar to other viral systems, some of these proteins are multitasking “swiss-army knife” proteins. For example, the Z protein of lymphocytic choriomeningitis virus (LCMV), in addition to its role in viral egress and budding, has been shown to interact with PML [8] to repress EIF4E-dependent translation [9,10], and to associate with the ribosomal protein P0 [11], although the functional relevance of this latter interaction has not yet been determined. 

Additionally, certain large-scale analyses of host proteomics [12,13,14], kinomics (study of phosphorylation changes) [15], transcriptomics [16,17,18], and metabolomics (changes in levels of small molecules) [19] have been undertaken to investigate the consequences of arenavirus infections. However, any resulting biological networks that were created only included the results of these individual studies. There is at present no integrated study linking all published findings relating to arenavirus-host interactions, and combining both the results of high throughput studies and more traditional single protein or pathway analyses. This review therefore seeks to summarize and organize current knowledge about arenavirus-host interactions in the context of a network, and to discuss current concepts in the analyses of such networks, in order to provide a “starter kit” systems biology reference as a supporting tool for arenavirus research. 

## 2. General Concepts in Systems Biology

### 2.1. Mathematical Concepts to Describe Biological Systems

A network is a collection of *nodes* or *vertices* connected by *edges*, which define pairwise relationships between the nodes. Networks are also referred to as *graphs*, with *graph theory *designating the mathematical field dedicated to the study of networks properties*.* Graph theory as a mathematical discipline is generally considered to date back to 1736, with the demonstration by Leonard Euler of the impossibility of finding a non-redundant path allowing the crossing of all seven bridges of Königsberg, which linked two islands to the rest of the city. By reformulating the problem in abstract terms, where land was represented as nodes and bridges as edges, Leonard Euler laid the foundations for graph theory [20]. However, despite attempts during the 20th century to introduce systems-level thinking into biology, notably with the work of Ludwig Von Bertalaffy [21,22], it is only in the early 2000’s with the development of high throughput molecular techniques, that systems biology has emerged as a significant field in biology. Attempts at modeling biological systems using existing mathematical tools revealed that, while some basic mathematical properties of networks could be ascribed biological relevance [23,24,25,26,27,28,29,30], novel analytic tools are needed in order to more aptly deal with the complexity of biological network architecture [31,32,33,34,35,36]. In this section, we will describe general network characteristics and their correlation to biological processes. For further information on systems-level thinking and mathematical concepts in systems biology, we refer the reader to the more comprehensive introductory books by Choi [37] and Alon [38].

### 2.2. Basic Principles in Biological Networks

**Figure 1:**
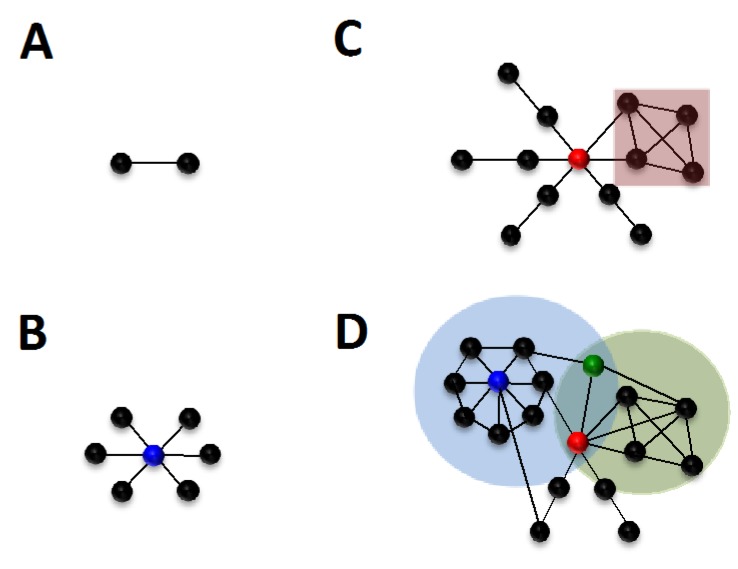
**Basic concepts in network analysis. **(**A**) Two nodes are connected by an edge. (**B**) One node is connected to several nodes, the blue color denotes the highest connectivity (*k*) value within the network. (**C**) The red node displays high centrality (*b*)**. **The red square designates a 4-node clique. (**D**) The network is more complex, the red node indicates the node with the highest centrality, but is not the node with the highest connectivity (blue node). The light green and blue circles show two communities in this network, and their shared nodes, the bridging node is shown as green.

In systems-level approaches to investigating cellular processes, the nodes within networks can represent any biological entity, including genes, proteins, complexes or small molecules. Edges represent the relationships between these entities, whether physical, for example receptor-ligand binding, or functional, such as the activation or inhibition of a given protein through phosphorylation (Figure 1A). A network can be composed of several *connected components*, *i.e*. groups of nodes can be connected to each other, but bear no common edges with the other subsets of nodes present within the network. A network is said to be fully connected if it is composed of a single connected component. Current knowledge of biological processes does not always allow the inclusion of specific nodes within the largest connected component of the network. These nodes may thus be found as *single nodes*, when no interaction has been characterized, or within a smaller connected component if information exists about their interaction with other biological elements, which also do not share an edge with any nodes of the largest connected component. It is important to note however, that this absence of connection of specific nodes to the largest connected component may not necessarily reflect biological properties, but rather may result from the lack of experimental data on the entity represented by that node [39].

### 2.3. Local Properties in Biological Networks

Local properties in biological networks refer to properties pertaining to a single node within the environment of its immediate neighbors. An elementary measure in networks is that of node *degree *or *connectivity* (*k*), which refers to the number of edges incident to that node (Figure 1B). Biological networks are usually inhomogeneous, and contain highly connected subnetworks [40], which can be defined at local or global levels. Locally, the *clustering coefficient* or *transitivity* defines the degree to which neighbors of a specific node are connected to each other [40,41]. A *clique*, or *complete graph*, designates a subset of nodes within the network which are fully connected, *i.e.* there is an edge connecting any two nodes within this subset [42] (Figure 1C). As such, the definition of cliques has been shown to strongly correlate with that of protein complexes or functional modules within networks [26].

### 2.4. Global Properties of Nodes and Edges

These properties describe information about the relative importance of network elements to the structure of the network. A common attribute within the network is the definition of the *shortest path *or *geodesic, *which is defined as the minimum distance between any two nodes in the network. From this the *mean path length* can be calculated by averaging all shortest paths for any two nodes in the network. In biological systems, path length can be used to understand the reactivity of pathways, for example a short path length within a signaling cascade ensures an efficient “information” flow, where only a small number of intermediate steps are required between the initial sensing of a given stimulus and the induction of a biological response, for example induction of gene expression [27]. 

Centrality or vertex betweenness (*b*) measures the number of shortest paths that pass through a given vertex (Figure 1C). This property theoretically describes the breadth of pathways a protein is involved with, and nodes with high scores are sometimes referred to as *bottlenecks*. *High-betweenness* or *bottleneck-ness* is a measure of the essentiality of a given protein to the biological system considered [28,29], and has been shown for protein-protein interaction networks to correlate with a regulatory function central to several pathways [30]. Centrality within the network can also be determined through edge betweenness, which calculates the number of shortest paths a specific edge participates in, and similarly to node betweenness, describes the essentiality of the biological relationship specified by the edge to the biological system considered.

### 2.5. Understanding Network Structure: Defining Communities

The goal of systems biology is to understand how the constitutive components of a network interact with each other. However, one of the main problem in systems biology is the mathematical characterization of such components in a way that is relevant to the elucidation of biological processes. In network theory, there are several ways to define subcomponents of a network, which we will refer to as *communities*, but which can also be designated as *clusters*, *cohesive groups* or *modules*. Most of the algorithms developed in graph theory rely on the definition of separated communities [43,44,45], which means that any node can only belong to one community. However, a biological element, for example a protein, can be part of several complexes or several pathways. In order to provide a better model for finding communities within biological networks, Palla and coworkers developed a novel algorithm, called the clique percolation method, which allows the identification of overlapping communities [31]. Analysis of a human infectome network using this algorithm revealed the unambiguous assignment of most communities to at least one cellular function [3], however reliable mathematical identification of complex biological associations, for example pathways, remains unresolved. Finally, the identification of shared nodes between communities also led to the characterization of *bridging* nodes, which, contrary to hubs which may be also shared by communities, display low local connectivity, but high centrality, and provide a link between one or more highly connected components [3] (Figure 1D).

## 3. Building and Analyzing an Arenavirus-Host Network

### 3.1. Datasets for the Construction of the Arenavirus-Host Network

Data pertaining to the arenavirus-host interplay was compiled from published studies on arenavirus-host protein-protein interactions [8,9,10,11,46,47,48,49,50,51,52,53,54,55,56,57,58,59,60,61,62], viral requirements for specific host factors [63,64,65,66,67,68,69,70,71,72,73,74,75,76,77,78,79,80,81,82,83], viral inhibition of host proteins [84,85,86], kinomics and proteomics studies of cellular changes in response to infection [12,13,14,15,87,88,89,90,91,92,93,94,95,96,97,98,99,100,101,102,103,104,105,106,107]. Data was also retrieved from studies describing co-localization of viral and host factors [108] or cellular factors that inhibit viral replication [109,110]. In total, 304 cellular proteins were identified as playing a role in arenavirus infection from this literature analysis (through September 2012). To simplify nomenclature, this primary set of 304 host proteins will be referred to as ‘host targets’ in this article. The proteins’ identities, as well as details of the nature of the virus-host interactions, and the specific viral systems in which the interaction was observed, are available in Table S1.

The usefulness of any network will of course only be as good as the primary data used to create it. Therefore, we have carefully curated the primary literature to establish that the experimental data reasonably supported any conclusions made in a publication about interactions, before including it in the dataset. However, we have not assigned any weight to a given interaction. Instead, details are provided that a user can access to determine for his or herself the strength of the data supporting a given interaction, and are available through the Description and References section within Table S1, or through the Description and Reference attribute within the network. In both cases, information is provided about whether the interaction was identified during virus infection, or through a sub-viral system such as the co-expression in cells of specific viral proteins.

**Figure 2:**
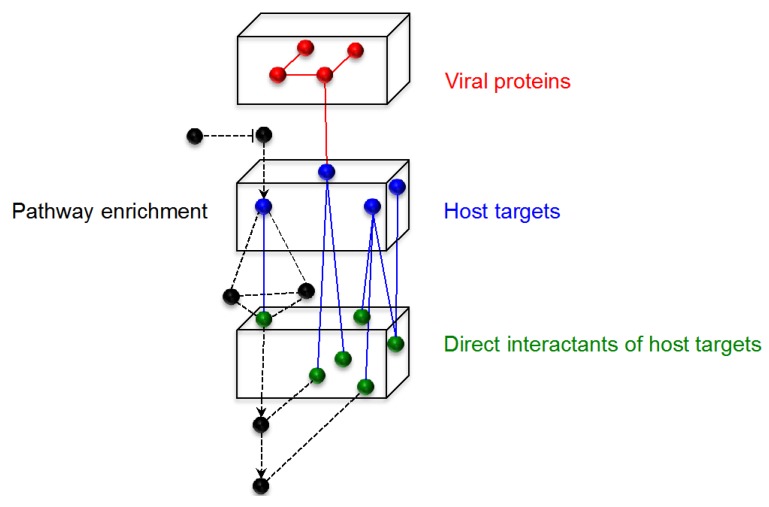
**Construction of the arenavirus-host network.** Schematic representation of steps in the construction of the arenavirus-host network. The four arenavirus proteins are represented by red nodes, host targets identified in the literature are blue nodes, and the directly interacting partners of these host targets, obtained from databases, are represented by green nodes. Black nodes represent proteins uploaded into the network through pathway enrichment performed on the host targets, and may contain, in addition to protein-protein interaction data, additional information about functional interactions between proteins. Edges indicate a relationship between two proteins, such as a direct protein-protein interaction or a process of protein modification, such as phosphorylation or ubiquitination.

A primary network was constructed, organized around the 4 arenaviral proteins and the 304 curated host targets. Protein-protein interactions and functional pathway information was then retrieved for each host target by searching the public databases available through Pathway Commons [111], and was merged with the primary network. Such functional enrichment of the primary network therefore allows a comprehensive overview of the host cell pathways affected by, or required during, arenavirus infection, based on current knowledge curated from the literature. A schematic of the network building process is shown in Figure 2, and the resulting arenavirus-host protein network is available as supplemental data (Supplemental Data—Arenavirus-Host Full Network.cys).

### 3.2. Programs for Building and Analyzing the Network

The arenavirus-host network was constructed in Cytoscape 2.8 [112,113], a freely available software for complex network visualization [114]. Retrieval of host protein-protein interactions, as well as pathway enrichment, was performed using the cPath software [115], which enabled access from the Cytoscape platform to the Pathway Commons Web Service [111], a web-based interface that allows the simultaneous mining of biological databases such as Reactome or BioGrid. Network visualization was also carried out in Cytoscape 2.8. Details on how to use the Cytoscape software, as well as links to advanced tutorials, are available in Supplemental File II.

General mathematical properties of the network, such as node connectivity and centrality, were determined using the R package igraph for network analysis [116]. A link to the igraph site, which includes information on how to download and use this software is provided in Supplemental File II.

### 3.3. Embedding Information (Attributes) in the Network

Any information regarding nodes and edges is encoded within the network as *attributes*. Different categories of information, such as gene or protein name, role in arenavirus infection, or the pathways the protein is active in, are encoded as individual attributes. For example the gene name of any given node can be found under the attribute “biopax.xref.GENE_SYMBOL”. This information can be retrieved within Cytoscape by directly clicking on the node or edge of interest, which will result in the display of attributes values in the data panel. These attributes can also be used to search for specific elements within the network, such as nodes involved in a given biological process or pathway. For example, the user can configure Cytoscape search options, by clicking on the icon immediately to the right of the search box, to be based on the attribute category “nodes.ListPathway”. The user can then type in the desired request in the search box, such as the term “translation”, which will highlight all nodes involved in this pathway within the network, and provide a list of these nodes in the data panel. Attributes can also be used to specify differential displays within the network (such as the node color or size, but also the layout of the selected nodes), or can be used to create subnetworks through the use of filters (discussed in Section 3.5).

### 3.4. Virus-Specific Elements Encoded within the Network

The arenaviruses are distinguished serologically as belonging to either the Old World (LCMV complex) or the New World (Tacaribe complex) viruses, with the New World viruses being further divided into clades, A, B and C. The members of these groups have both common and distinct features, and pathogenic and non-pathogenic viruses are present in both of the two major serological complexes. The network we built includes all relevant information about the arenavirus-host relationships that we obtained from the literature, and includes observations about the following viruses: Old World (LASV, LCMV, MOPV, MOBV), New World clade A (PICV), clade B (JUNV, MACV, GTOV, AMAV, TCRV, SABV), clade C (OLVV, LATV), and clade A/B (WWAV). The network also contains information, where available, about different strains of viruses used, with human pathogenic strains denoted with a subscript ‘v’ and non-pathogenic strains with the subscript ‘a’, for example the attenuated PICV variant P2 is denoted PICV_a_, and the virulent PICV variant P18 is denoted as PICV_v_ [117,118]. The information for each investigated virus is reported as string values (TRUE/ FALSE/ Not tested), indicating whether the interaction or the node has been confirmed for the specific virus indicated, or if no studies have yet evaluated the role of a host protein interaction for that arenavirus family member. 

Additional information linking the interaction to a summary of findings, and a reference to the original published report for a specific virus-host relationship, is also accessible within the network through the attribute “nodes.Description and References”.

### 3.5. Using Filter-Set Subnetworks

The advantage of building a full network, that includes information from all published reports, is that the information pertaining to all arenaviruses is encoded in one single file. This full or master network should be considered a work in progress, and future experimental data will both confirm and add to the value of the relationships embedded in the present version (September 2012). The network is also paradoxical, since it includes data that is both true and false for different viral systems. For example, the binding of GP to two known receptors for the arenaviruses, human transferrin receptor 1 and alpha-dystroglycan [50,51,52,53,54,55,56,57], is both true and false, depending on the virus strain. Therefore, in order to perform relevant systems-level analyses for the arenaviruses, the network should be customized through the use of filters, from which a relevant subnetwork can be derived through user-driven curation, and depending on investigative needs. 

Filters can be set within from within the Cytoscape software. The available filters can be applied to any attribute that is assigned to either nodes or edges (for example, to select only viral host targets), as well as topology (for example, to select neighbors at a given distance from nodes of interest). Multiple filters can be combined through the use of Boolean links: AND (–node or edge selected must pass both filters), OR (–node or edge selected must pass at least one filter) and NOT (–to exclude nodes or edges). 

As a practical example, if a user wanted to generate a network displaying only the proteins that have been shown to play a role during infection by the pathogenic strains of JUNV, the following filter would be applied: nodes. JUNV_v_ > TRUE. However, since this filter will only select a subset of the host target proteins, information and context could be lost. To put this data into a more relevant context, we have encoded a “Connect pathways” attribute. In this case, the user can select both the subset of viral host targets that are specific to pathogenic JUNV strains, as well as maintaining the integrity of the pathways that these proteins are involved in, by applying the following filter: nodes.Connect pathways >JUNV_v_. Finally, the specific subnetwork can be created and saved as a separate network through the path: File > New > Network > From selected nodes, All edges, and further analyzed using Cytoscape Network Analyzer, or other network analysis tools such as igraph. 

### 3.6. Identifying Potential Viral Targets Through Centrality and Connectivity Values

**Figure 3:**
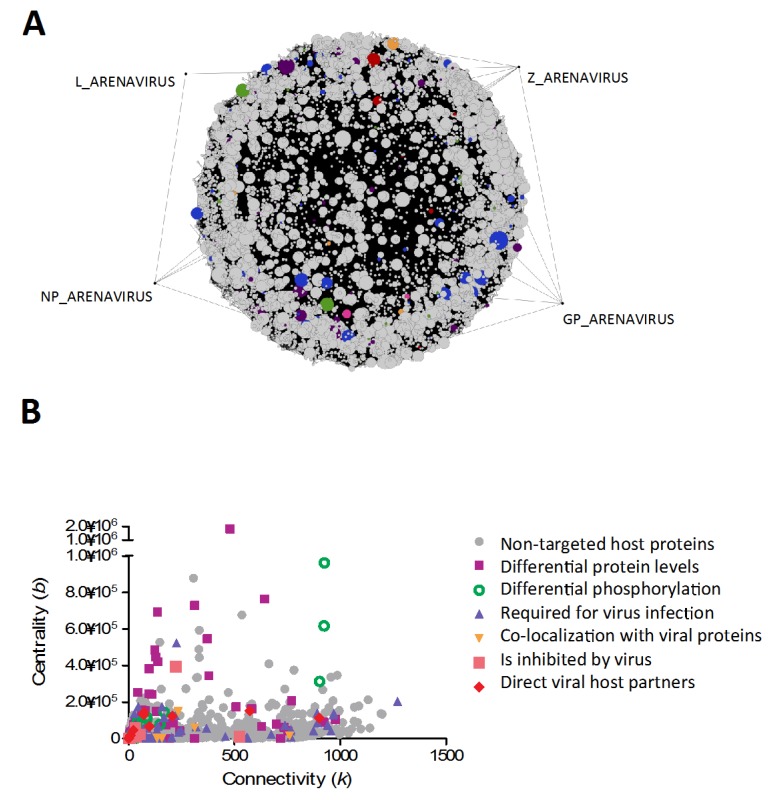
Common element network of arenavirus-host interactions, describing pathways targeted/perturbed during infection by all arenaviruses. (**A**) In this graphical representation, the arenaviral proteins (GP, NP, L and Z) are highlighted by dragging out of the main connected components, to facilitate visualization. Nodes are color-coded depending on role during arenavirus infection (see key in part b). Node size was correlated to node degree, to facilitate visualization of their relative importance within the network. Interested users can find the filter used to generate this network pre-loaded within the supplemental network file (Supplemental Data: Arenavirus-Host Full Network.cys; Filter name: Common elements of arenavirus replication).(**B**) Graphical representation of characteristics of nodes (connectivity and centrality values). Nodes of particular importance are revealed by high values in both parameters. The nodes are color-coded depending on their identified role during virus infection.

Using the tools described above, we generated a subnetwork to investigate common viral host targets in arenavirus infection, and to analyze the role of these proteins in the context of the pathways they contribute to. As highlighted previously, viruses tend to target highly connected nodes within networks [3,4]. This can explain how viruses expressing only a small subset of proteins can subvert major cellular pathways, through the strategic targeting of these central elements. In order to identify potential hubs within the network, we plotted centrality values *vs* connectivity values for all nodes present in the common subnetwork (Figure 3B). The node with the highest connectivity value was found to be p53, a master regulator of cellular processes, whose steady-state levels are decreased during arenavirus infection [15], although no mechanism for this observation has yet been identified. Other hubs, which have already been identified as arenaviral host targets, include nucleophosmin [15], involved in ribosome biogenesis, and cytoskeletal elements such as vimentin [15], tubulin [70,71,72,77] and actin [13,70,71,72]. Amongst hub proteins that have not been previously identified as viral host targets, we found general adapters in signal transduction pathways such as YWHAZ or SHC1, as well as the master regulator AKT1 kinase, which is involved in many cellular processes such as metabolism, proliferation, cell survival and growth, and angiogenesis. These provide interesting leads for further investigation, since any direct effect on these proteins could potentially explain downstream phenotypes previously reported. 

## 4. Biological Networks and Arenavirus Pathogenesis

Despite the severity of the symptoms resulting from infection by pathogenic arenaviruses, which include hemorrhagic fevers and/or severe neurological symptoms, very limited tissue damage is typically observed in the vascular endothelium, brain, or other organs [119]. It is at present not clear what causes this significant morbidity, with the current hypothesis being that these effects are the consequences of as-yet-undescribed changes in the infected cell [120]. Thus, analysis of arenavirus-host networks may yield insights into the underlying basis of pathogenicity, by suggesting leads for further investigation. 

For example, analysis of an arenavirus network, built around gene products differentially expressed in virulent and avirulent LCMV infections of rhesus macaques, highlighted a potential role for the epidermal growth factor receptor EGFR in infection by pathogenic arenaviruses [17]. Furthermore, EGFR also displayed different patterns of phosphorylation in a kinomics comparison of pathogenic and non-pathogenic PICV infections, which was correlated with activation or inhibition of its receptor activities [15]. EGFR is involved in signaling cascades that lead to a wide array of cellular changes such as cytoskeletal rearrangement, changes in gene expression, anti-apoptosis and increased cell proliferation, and it will be of great interest to elucidate its potential role in arenavirus pathogenesis.

## 5. Convergence Between Different Virus-Host Networks

Systems biology can also be used to compare different viral-host systems, in an attempt to identify common targets of viral infection, and thus highlight convergent mechanisms of viral pathogenesis. For example, Bowick and McAuley reported on a systems-level meta-analysis of high-throughput datasets from hemorrhagic fever systems of various viral aetiologies [121]. The data analyzed included proteomics studies of responses to PICV infection [14] and transcriptomics analyses carried out during LCMV infection [16,17], both of which are used as models for Lassa fever, as well as microarray analyses from heterologous viral systems such as the filovirus Ebola virus (EBOV) [122], and bunyavirus Rift Valley Fever virus (RVFV) [123]. This analysis resulted in the identification of cyclooxygenase-2 (COX-2) as a common viral target, which was downregulated during LCMV and RVFV infections, but upregulated during infection by EBOV [121]. As noted by the authors, this result can further be extended to infection by the flavivirus Dengue virus, which was found to induce COX-2 expression [124]. COX-2 catalyzes the production of prostaglandin precursors, which are subsequently converted to active prostaglandin molecules such as prostacyclin PGI_2_, a vasodilator, by tissue-specific isomerases [125]. This finding of convergent targeting of prostaglandin pathways by hemorrhagic fever viruses from different families suggests an exciting new area of research towards unraveling the basis of hemorrhagic fever syndromes.

Systems-level combining of virus-host networks of different viral families has also been undertaken in recently published studies [3,126]. One study investigating common host targets of 70 viral proteins from 30 viruses uncovered the ubiquitous viral targeting of hnRNPU, phosphatidylinositol-3 OH kinase (PI3K), the WNK kinase family, and the ubiquitin-specific peptidase 19 [126]. Interestingly, PI3K has already been identified as essential for arenavirus infection [66,67,68], however, a direct interaction of PI3K with a viral partner has not yet been demonstrated. Moreover, hnRNPU, USP19 and WNK kinases 1 and 4 are also present in the arenavirus-host network, indicating their involvement in pathways identified for viral host targets.

In an another study, Navratil and coworkers developed a model containing virus-host interactions for 110 viruses from 8 different viral families, constituting the most comprehensive pan-viral “infectome” network available at present. Further annotation of host proteins regarding their known involvement in diseases revealed the significant association of 57 viruses with 34 diseases [3]. These studies are part of a current trend to investigate convergence in virus-host networks, and establish links through systems-level analysis between molecular characteristics and pathogenesis. One goal of these approaches is to generate a global viral infectome, with which to model general characteristics of the infected cell, and thereby identify suitable targets for the development of pan-viral therapeutic strategies.

## 6. Systems Biology to Suggest Therapeutic Targets

Therapeutic strategies that target cellular processes essential for virus infection are attractive since they are less likely to result in viral evolution towards drug-resistance [127]. In this way, biological networks can be used to identify candidate targets based on the mathematical properties of specific nodes. In network theory, situations where a small subset of proteins have a large number of interactions, while the majority of nodes have lower connectivity within the network, correlate with robustness against random attacks, which is characterized by the removal of any node or edge from the network. This robustness can be explained by the lower probability for a node with a higher number of interactions to be targeted in random attacks, meaning that the overall connectivity of the network is conserved. Analyses of these properties can be harnessed in biological networks towards the determination and testing of therapeutic targets *in silico *- effectively simulating an ‘attack’. In addition to hubs, bridging nodes (lower connectivity but connecting highly connected components), should be considered in attack analyses, since their removal could result in disconnection of the network. 

Network analysis of large-scale RNAi screens has been used to identify a correlation between the preference for viruses to interact with highly connected host proteins and the functional essentiality of these proteins in virus infection [3]. Furthermore, amongst the lower-connected proteins represented in those screens, a predominance of bridging elements was observed. Interestingly, when targeting bridging nodes within the network, a lower impact on network topology was observed than when targeting central nodes [3]. It has been argued that targeting bridging proteins instead of highly connected nodes might result in lower toxicity within the host [3,128,129]. While it is true that extensive network disruption is likely to be detrimental to the host, only empirical testing will truly determine drug toxicity *versus* efficacy, and indeed whether a temporary toxicity can be tolerated in an attempt to thwart a severe viral disease such as the arenviral hemorrhagic fevers. 

Systems biology is also used in pharmacogy for drug discovery or drug repositioning. By network analysis of phenotypic side-effect similarity of chemically dissimilar drugs, a recent demonstration was made of the power of systems biology to uncover shared cellular targets, and therefore potential alternative applications for existing drugs [130]. As networks grow in sophistication and power, reliable systems-level models of the infected cell or organism might not be so far ahead. And since the pathogenic arenaviruses require BSL4 containment, the pre-screening, *in silico*, of the antiviral potential of existing drugs appears all the more an attractive option.

## 7. Conclusions

Systems biology has been readily embraced by virologists, with the creation of databases entirely devoted to virus-host relationships such as VirhostNet [131] or VirusMint [132], as well as virus-specific ones such as HCVpro [133], and with the development of increasingly comprehensive networks being developed for several viruses [3,134,135]. In this study, we provide the first comprehensive synthesis of all published accounts of arenavirus-host protein interactions (September 2012) and have used this information to build a “starter kit” arenavirus-host network. It is expected that future unbiased studies of protein-protein interactions, including proteomics screens, will significantly improve its accuracy.

As -omics data become more abundant and refined, the next challenge for systems biology will be to integrate datasets pertaining to different biological entities (small molecules, proteins, genes) into a single network. At the same time, modeling of cellular processes, as well as viral infection, will need to take into account spatial (subcellular localization) and temporal (time post infection) parameters, in order to generate dynamic networks that more accurately reflect cellular processes. Also, in the (not-so-distant?) future, it might be possible to generate a reliable *in silico *model of arenavirus infection.

## Supplementary Files

Supplementary File 1

Supplementary File 2
