# Supplementary material for: A Systems Biology Starter Kit for Arenaviruses
Source: Viruses. 2012 Dec 11;4(12):3625–46. doi: 10.3390/v4123625 (PMC3528283; doi:10.3390/v4123625)
Supplement: Supplementary File 2 [file viruses-04-03625-s002.pdf]

Supplemental File II

Instructions for Accessing and Analyzing the Arenavirus-Host Network

1. Download the Cytoscape software at www.cytoscape.org/download.html. You will need to agree to licensing terms, and register prior to download, and the Cytoscape software is free of charge.

2. Once the software is installed, open the Cytoscape application file.

3. You may now access the Arenavirus-Host network by clicking File>Open>Arenavirus-Host full Network.cys (Supplemental File III to this manuscript).

4. Once the file is loaded, a visualization of the network will appear in the central part of the screen. Tabs within the **Control panel** on the left include a general description of the network (Network), a tool to change visualization parameters of the network (VizMapper™), a tool to add nodes or edges (Editor), and a tool to select/deselect nodes and edges according to specific attributes (Filters).

On the bottom part of the screen is the **Data panel**, which allows visualization of the nodes/edges/network attributes. An element needs to be selected in order to retrieve its attributes, done either by directly clicking on a node or edge within the visualized network, or using the search toolbar at the top of the screen, or by setting up filters.

Attributes listed include node label gene symbol. In addition, for the direct host targets that have been curated from the literature, information is also available on the nature of the published interaction with the arenaviruses. Pathway information was encoded using the ListPathway attribute, and specific pathways can be selected using the search option, or through the use of filters. The list of attributes displayed can be modified using the icons present at the top of the Data panel.

List of nodes and edges, as well as their attributes can be exported by right-clicking on the list, and selecting the Export option. The resulting file can be then imported into Microsoft Excel or opened through a text editing software.

5. Tools included in the Cytoscape software include network analysis tools, and display tools. In order to further analyze the network, the user can use Plugins>Analyze Network to perform standard calculations on the network such as degree or clustering coefficient. Parameters for such calculations can be changed through the Plugins>NetworkAnalyzer settings. A wide range of advanced tools to analyze or display the network are also available as plug-ins from http://apps.cytoscape.org

6. Further information on how to use Cytoscape, as well as release-specific issues can be found at http://www.cytoscape.org/documentation_users.html

7. As the network size increases, it may become necessary to use another application to perform calculations on this network. In that configuration, we recommend the use of igraph or similar network analysis package. Download information and detailed instructions for the igraph package are available at: http://igraph.sourceforge.net

8. In order to identify potential functional clusters within the network, communities can be calculated by using different algorithms, as explained in section 2.5 of the manuscript. The igraph package can be used to identify non-overlapping communities, whereas the CFinder software can be used to identify overlapping communities, and can be downloaded at: http://hal.elte.hu/cfinder/
